# 探究单孔胸腔镜应用于高龄非小细胞肺癌患者的临床疗效：肺叶、肺段及两者对比分析

**DOI:** 10.3779/j.issn.1009-3419.2018.04.11

**Published:** 2018-04-20

**Authors:** 麟 黄, 斌 郑, 椿 陈, 炜 郑, 勇 朱, 朝晖 郭

**Affiliations:** 350001 福建，福建医科大学附属协和医院 Department of Thoracic Surgery, Fujian Medical University Affiliated Union Hospital, Fuzhou 350001, China

**Keywords:** 单孔胸腔镜, 高龄, 肺肿瘤, 肺叶切除术, 肺段切除术, Single-port video-assisted thoracoscopy, Elderly, Lung neoplasms, Lobectomy, Segmentectomy

## Abstract

**背景与目的:**

肺癌的发病率长期位于癌症之首。Ⅰ期、Ⅱ期和部分Ⅲ期非小细胞肺癌(non-small cell lung cancer, NSCLC)的主要治疗方式以手术为主，肺叶切除术与肺段切除术为两类较为常见的手术方式。电视辅助胸腔镜手术(video-assisted thoracoscopic surgery, VATS)已广泛应用于临床，单孔电视辅助胸腔镜(single-port video-assisted thoracoscopic surgery, SP VATS)在胸部外科手术中的应用也逐渐被国内外专家所认识和接受。随着社会高龄化程度逐渐加深，这类患者成为NSCLC诊疗的难点。本研究探讨并分析SP VATS肺叶切除术与肺段切除术在治疗高龄NSCLC患者中的临床应用价值。

**方法:**

回顾性分析福建医科大学附属协和医院胸外科在2014年5月-2016年12月期间行SP VATS肺叶切除与肺段切除的417例患者资料。其中高龄患者139例(肺叶切除124例*vs*肺段切除15例)，非高龄患者278例(肺叶切除248例*vs*肺段切除30例)。分别比较高龄与非高龄患者行SPVATS肺叶或肺段切除术及高龄患者行肺叶、肺段切除术的围手术期及术后短期恢复情况。

**结果:**

SP VATS肺叶切除和肺段切除的比较组中，除在术前合并症患病率上，高龄患者均高于非高龄患者(*P* < 0.05)，在其余比较项上无明显差异(*P* > 0.05)。在单孔胸腔镜肺叶切除和肺段切除的高龄患者比较中，可发现淋巴结清扫站数[(7.61±0.21)组*vs* (5.60±0.35)组]及数目[(20.39±0.97)枚*vs*(15.40±2.64)枚]，差异有统计学意义(*P* < 0.05)，而在年龄、术前合并症患病率、平均手术时间、术中失血量却无统计学差异(*P* > 0.05)。虽然在术后并发症发生率上肺叶切除和肺段切除的高龄患者无统计学差异(*P* > 0.05)，但是在术后房颤和双下肢静脉血栓发生率上却存在明显差异(*P* < 0.05)。在术后住院时间[(3.18±1.32)天*vs* (5.04±1.30)天]、胸管放置时间[(7.00±1.31)天*vs* (5.00±0.74)天]及总住院费用[(70.06±5.23)千元*vs* (61.20±5.22)千元]上，差异无统计学意义(*P* > 0.05)。

**结论:**

高龄患者由于合并更多基础疾病，可能增加术后并发症的风险，但单孔胸腔镜肺叶及肺段切除术并不增加高龄患者的手术相关风险，且对合适的高龄病例行肺段切除可获得与肺叶切除相类似的短期疗效。

## Abstract (en)

**Background and objective:**

The morbidity of lung cancer has long been the highest in cancer.Stage Ⅰ, stage Ⅱ and partly of stage Ⅲ non-small cell lung cancer (NSCLC) are mainly treated by surgery.Lobectomy and segmentectomy both are common lung resection methods.Video-assisted thoracoscopic surgery (VATS) has been widely used in clinical, and the application of single-portvideo-assisted thoracoscopic surgery (SP VATS) has gradually been recognized and accepted by professors.With increasing degree of eldly in society, eldly patients already have become inceasingly difficulties in the diagnosis and treatment of NSCLC.The aim of this study is to explore and analyze clinical value of SP VATS lobectomy and segmentectomy in elderly patients with NSCLC.

**Methods:**

In this retrospective observational study, the outcomes of 417 consecutive patients who had undergone SP VATS anatomic segmentectomy or lobectomy for NSCLC from May 2014 to December 2016 on department of thoracic surgery in Fujian Medical University Affiliated Union Hospital were examined, including 139 elderly-case (lobectomy *vs* segmentectomy: 124 *vs* 15) and 278 nonelderly-case (lobectomy *vs* segmentectomy: 248 *vs* 30).The condition of perioperative period and postoperative short-time recovery could be compared with lobectomy and segmentectomy between elderly and nonelderly cases and lobectomy and segmentectomy in elderly cases.

**Results:**

The morbidty of preoperative complications was significant difference (*P* < 0.05) in comparing with elderly and non-elderly patients with NSCLC either in SP VATS anatomic segmentectomy orlobectomy, except others is no significant difference (*P* > 0.05).Numbers of dissected lymph nodes and mediastinal nodal stations of SP VATS lobectomy in elderly patients with NSCLC were more than segmentectomy (*P* < 0.05), which were (7.61±0.21) *vs* (20.39±0.97) and (5.60±0.35) *vs* (15.40±2.64).But there was not significant difference between two elderly groups of SP VATS lobectomy and anatomic segmentectomy in age, morbidty of preoperative complications, average operation time and intraoperative blood loss (*P* > 0.05).Postoperative drainage volume [(1, 150.15±140.02) mL *vs* (853.53±177.04) mL] and duration [(7.00±1.31) d *vs* (5.00±0.74) d], duration of postoperative hospital stay [(3.18±1.32) d *vs* (5.04±1.30) d], costs [(70.06±5.23) thousands yuan *vs* (61.20±5.22) thousands yuan ] or postoperative complications (5.97% *vs* 20.00%)(*P* > 0.05).Notwithstanding, group of SP VATS anatomic segmentectomy found more postoperative atrialfibrillation and the vein thrombosis of lower limbs (*P* < 0.05).

**Conclusion:**

Elderly patients having more basic diseases and taking increasely risk of postoperative complications.SP VATS anatomic segmentectomy andlobectomy do not increase the risk of elderly patients with surgery.SP VATS segmentectomy is as safe and effective as SP VATS lobectomy in elderly patients, who fit to undergo SP VATS segmentectomy seem to get as same short-time effect as SP VATS lobectomy.

近年来，肺癌的发病率长期位于癌症之首，我国也有类似现象^[1-3]^。目前Ⅰ期、Ⅱ期和部分Ⅲ期NSCLC(non-small cell lung cancer, NSCLC)的主要治疗方式以手术为主^[4, 5]^——肺叶切除及纵隔淋巴结清扫术被认为是早期NSCLC的标准治疗方法。早在1995年开始，Ginsberg等^[6]^认为亚肺叶切除是仅适用于高龄且肺功能不全的肺癌患者的一种妥协性术式的研究结果，美国国立综合癌症网络(National Comprehensive Cancer Network, NCCN)指南及我国原发性肺癌诊疗规范均对亚非叶切除的适应症做了相关叙述^[7-10]^。电视辅助胸腔镜手术(video-assisted thoracoscopic surgery, VATS)已广泛应用于临床，单孔电视辅助胸腔镜(single-port video-assisted thoracoscopic surgery, SP VATS)在胸部外科手术中的应用也逐渐被国内外专家所认识和接受^[11-14]^。高龄NSCLC患者基础疾病较多，早期难于与自身的呼吸系统慢性疾病相鉴别，易耽误就诊时机，术后机体恢复慢，具有易多虑、易自我放弃等特殊心理特点，成为治疗难点。

目前关于单孔胸腔镜运用于高龄肺癌患者的研究相对较少，更没有关于单孔全胸腔镜肺叶切除术与肺段切除术对于高龄NSCLC患者临床疗效的比较分析研究。因此，本研究旨在探讨并分析单孔胸腔镜肺叶切除术与肺段切除术在治疗高龄NSCLC患者中的临床应用价值。

## 1 资料与方法

### 1.1 一般资料

#### 1.1.1 研究对象

福建医科大学附属协和医院胸外科从2014年5月-2016年12月成功对NSCLC患者实行单孔胸腔镜肺叶切除与肺段切除共550例，其中肺叶切除422例，肺段切除128例。依据纳入标准和排除标准，选取符合本研究入组患者。

#### 1.1.2 术前检查

术前疑诊肺部肿瘤的患者均应完成术前血液学、影像学及纤维支气管镜评估。必要时行全身正电子发射型计算机断层显像(positron emission computed tomography, PET)以评估是否存在远处转移及有无手术指征。

#### 1.1.3 病例纳入标准

① 行肺叶、肺段切除+系统性纵隔淋巴结清扫术或采样术的病例符合第七版国际抗癌联盟(Union for International Cancer Control, UICC)NSCLC分期标准及2015版NCCN NSCLC临床实践指南推荐的手术适应证；②术前排除远处转移可能，未行放、化疗且心肺功能可耐受手术；③术中冰冻切片证实为微浸润性腺癌、浸润性腺癌或鳞状细胞癌；④单孔全胸腔镜肺叶切除术或解剖性肺段切除术由具有丰富经验及常规行胸腔镜或开胸手术的固定团队进行；⑤高龄组为年龄≥65岁的NSCLC患者，非高龄组为年龄 < 65岁的NSCLC患者^[15]^。

#### 1.1.4 病例排除标准

① 术前相关检查提示心肺功能无法耐受全身麻醉手术方式；②合并其他恶性肿瘤病(史)；③术前有新辅助放、化疗或放化疗治疗史；④术前影像学提示为多发肺结节，手术涉及不同的肺段、肺叶处理或肺段切除加肺叶切除；⑤术中病理证实为以下病变(支气管扩张症、炎性假瘤、结核性肉芽肿、硬化性血管瘤、不典型腺样增生，肺转移瘤等)，未进行系统性纵隔淋巴结清扫术或采样术；⑥术中病理证实胸膜转移；⑦术中发现胸膜腔广泛粘连的患者。

### 1.2 研究方法

采用回顾性分析的方法，依据标准选取单孔胸腔镜肺叶切除高龄组(SP VATS-LE组)124例、单孔胸腔镜肺段切除高龄组(SP VATS-SE组)15例，再选取单孔胸腔镜肺叶切除非高龄组(SP VATS-LNE组)248例、单孔胸腔镜肺段切除非高龄组(SP VATS-SNE组)30例，共417例病例，形成3对比较组，即SP VATS-LE组*vs* SP VATS-LNE组，SP VATS-SE组*vs* SP VATS-SNE组，SP VATS-LE组*vs* SP VATS-SE组，对临床病例资料中的各项指标进行比较分析。手术时间定义为麻醉开始至麻醉复苏，包括术中快速冰冻检查时间及肺楔形切除操作时间，术中出血量统计止血纱布及吸引管总和，围手术期死亡的定义是在住院期间或手术后30 d内发生的死亡。肺部感染定义为痰培养找到细菌或真菌病原学证据。心脏相关合并症包括先天性或后天性心脏病、心律失常、传导阻滞、冠心病等；肺部合并症包括慢性阻塞性肺疾病(chronic obstructive pulmonary disease, COPD)、肺炎、支气管扩张等。

### 1.3 统计学方法

统计分析采用SPSS 20.0软件进行。连续变量表示为均数±标准差(Mean±SD)，应用*LSD*-*t*检验的方法来比较是否有统计学差异。用*Fisher*精确检验或卡方检验对分类变量进行分析。*P* < 0.05为差异有统计学意义。

## 2 结果

### 2.1 患者的一般临床资料

回顾选取入组的417例患者相关临床资料，其中高龄患者139例(肺叶切除组124例/肺段切除组15例)，非高龄患者278例(肺叶切除组248例/肺段切除组30例)，男性160例，女性257例，平均年龄(58.44±0.53)岁。在肺叶切除组和肺段切除组中，高龄患者与非高龄患者在性别及肿瘤位置上无明显统计学差异(*P* > 0.05)。比较肺叶切除和肺段切除的高龄患者，在性别和年龄上的差异无统计学意义(*P* > 0.05)。各组一般临床人口统计学资料比较分析具体见表 1、表 2、表 3。

**1:** SP VATS-LE与SP VATS-LNE一般临床资料[*n*(%)] Clinical general condition of SP VATS-LE and SP VATS-LNE [*n*(%)]

	SP VATS-LE (*n*=124)	SP VATS-LNE (*n*=248)	*χ*^2^	*P*
Gender			3.52	0.06
Male	57 (45.97)	89 (35.89)		
Female	67 (54.03)	159 (64.11)		
Tumor Iocation			7.64	0.11
Left upper lob	20 (16.13)	21 (8.47)		
Left lower lob	18 (14.52)	58 (23.39)		
Right upper lob	41 (33.06)	79 (31.85)		
Right middle lob	13 (10.48)	25 (10.08)		
Right upper lob	32 (25.81)	65 (26.21)		
SP VATS-LE: the elderly group of lobectomy in single-port video-assisted thoracoscopic surgery; SP VATS-LNE: the nonelderly group of lobectomy in single-port video-assisted thoracoscopic surgery.				

**2:** SP VATS-SE与SP VATS-SNE一般临床资料[*n*(%)] Clinical general condition of SP VATS-SE and SP VATS-SNE [*n*(%)]

	SP VATS-SE (*n*=15)	SP VATS-SNE (*n*=30)	*χ*^2^	*P*
Gender			0.829	0.362
Male	6 (40.00)	8 (26.67)		
Female	9 (60.00)	22 (73.33)		
Tumor Iocation			12.78	0.385
LS1+2	4 (26.67)	3 (10.00)		
LS1+2+3	2 (13.33)	3 (10.00)		
LS4+5	0 (0.00)	1 (3.33)		
LS6	1 (6.67)	2 (6.67)		
LS8	1 (6.67)	1 (3.33)		
RS1	4 (26.67)	5 (16.67)		
RS1+2	0 (0.00)	2 (6.67)		
RS1+3	0 (0.00)	1 (3.33)		
RS2	0 (0.00)	2 (6.67)		
RS3	0 (0.00)	3 (10.00)		
RS6	1 (6.67)	6 (20.00)		
RS7+8	0 (0.00)	1 (3.33)		
RS8	2 (13.33)	0 (0.00)		
SP VATS-SE: the elderly group of anatomic segmentectomy in single-port video-assisted thoracoscopic surgery; SP VATS-SNE: the nonelderly group of anatomic segmentectomy in single-port video-assisted thoracoscopic surgery; LS1+2: left upper lob of apicodrsale segment; LS1+2+3: left upper lob apicodrsale and ventral segment; LS4+5: left upper lob of lingularsegment; LS6: left lower lob of superius segment; LS8: left lower lob of ventrobasale segment; RS1: right upper lob ofapicalissegment; RS1+2: right upper lob of apicalis and dorsalis segment; RS1+3: right upper lob of apicalis andventralis segment; RS2: right upper lob of dorsalis segment; RS3: right upper lob ofventralis segment; RS6: right lower lob of superius segment; RS7+8: right lower lob of mediobasale and ventrobasale segment; RS8: right lower lob of ventrobasale segment.				

**3:** SP VATS-LE与SP VATS-SE一般临床资料[*n*(%)] Clinical general condition of SP VATS-LE and SP VATS-SE [*n*(%)]

	SP VATS-LE (*n*=67)	SP VATS-SE (*n*=15)	*χ*^2^	*P*
Gender			1.93	0.17
Male	40 (59.71)	6 (40.00)		
Female	27 (40.29)	9 (60.00)		
Age (Mean±SD, yr)	69.72±0.49	71.00±1.16	1.09	0.28
SP VATS-LE: the elderly group of lobectomy in single-port video-assisted thoracoscopic surgery; SP VATS-SE: the elderly group of segmentectomy in single-port video-assisted thoracoscopic surgery.				

### 2.2 患者术前合并症发生率及肺功能

比较肺叶切除两组病例术前合并症发生率及肺功能，高龄组比非高龄组患者术前合并症发生率高(*P* < 0.05)。在高血压病和糖尿病上，高龄组患病率较非高龄组高(*P* < 0.05)。在术前肺功能上两组并无明显统计学差异(*P* > 0.05)。比较肺段切除两组病例肺功能有类似肺叶切除两组的结果(*P* > 0.05)。在术前合并症中，总体发生率上高龄组较非高龄组高，除肺部合并症和心脏相关合并症两个观察项目上患病率无统计学差异(*P* > 0.05)，其余项上高龄组患病率均高于非高龄组(*P* < 0.05)。比较肺叶切除高龄组与肺段切除高龄组术前合并症发生率及肺功能无明显差异(*P* > 0.05)。具体见表 4。

**4:** 术前合并症发生率及肺功能[*n*(%)] The morbidity of preoperative complications and lung function [*n*(%)]

	SP VATS-L (*n*=372)		*P*	SP VATS-S (*n*=45)		*P*	SP VATS-E (*n*=139)		*P*
	E (*n*=124)	NE (*n*=248)		E (*n*=15)	NE (*n*=30)		L (*n*=67)	S (*n*=15)	
Preoperative complications	71 (57.26)	83 (33.48)	0.00	9 (60.00)	5 (16.67)	0.00	37 (55.22)	9 (60.00)	0.74
Lung complications	12 (9.68)	16 (6.45)	0.27	2 (13.33)	2 (6.67)	0.50	7 (10.45)	2 (13.33)	0.75
Hypertension	53 (42.74)	38 (15.32)	0.00	8 (53.33)	1 (3.33)	0.00	26 (38.81)	8 (53.33)	0.30
Diabetes	16 (12.90)	16 (6.45)	0.04	4 (26.67)	1 (3.33)	0.02	12 (17.91)	4 (26.67)	0.44
Heart complication	14 (11.29)	19 (7.66)	0.25	3 (20.00)	1 (3.33)	0.06	12 (17.91)	3 (20.00)	0.85
FEV1%	88.67±1.26	89.41±0.82	0.61	85.51±2.44	89.93±1.91	0.18	89.83±1.44	85.51±2.45	0.19
SP VATS-L: the group of lobectomy in single-port video-assisted thoracoscopic surgery; SP VATS-S: the group of segmentectomy in single-port video-assisted thoracoscopic surgery; SP VATS-E: the elderly group of single-port video-assisted thoracoscopic surgery; E-the elderly group; NE: the nonelderly group; L: the lobectomy group; S: the segmentectomy group; FEV1%: FEV1/FVC; FEV1: forced expiratory volume in one second; FVC: forced vital capacity.									

### 2.3 术后病理资料

肺叶切除高龄组与非高龄组术后常规病理示切除病灶最大径分别为(2.28±0.10)cm和(1.91±0.08)cm，肺段切除高龄组与非高龄组术后常规病理示切除病灶最大径分别为(0.82±0.07)cm和(0.80±0.06)cm，差异均无统计学意义(*P* > 0.05)。然而，高龄患者中行肺叶切除较肺段切除者最大径更长，体积更大(*P* < 0.05)，常规术后病理示浸润性腺癌和鳞状细胞癌多(*P* < 0.05)。具体各组术后病理资料对比分析见表 5。

**5:** 术后病理资料[*n*(%)] Postoperative pathological data [*n*(%)]

	SP VATS-L (*n*=372)		*P*	SP VATS-S (*n*=45)		*P*	SP VATS-E (*n*=139)		*P*
	E (*n*=124)	NE (*n*=248)		E (*n*=15)	NE (*n*=30)		L (*n*=67)	S (*n*=15)	
The largest diameter of tumor (Mean±SD, cm)	2.28±0.10	1.91±0.08	0.15	0.82±0.07	0.80±0.06	0.46	1.79±0.09	0.82±0.07	0.00
Pathologic types			0.00			0.82			0.00
Microinvasive adenocarcinoma	12 (9.68)	57 (21.77)		10 (66.67)	21 (70.00)		10 (14.93)	10 (66.67)	
Invasive adenocarcinoma	100 (80.64)	180 (73.79)		5 (33.33)	9 (30.00)		48 (71.64)	5 (33.33)	
Squamous-cell carcinoma	12 (9.68)	11 (4.44)		0 (0)	0 (0)		9 (13.43)	0 (0)	
Pathologic stage			0.09			1.00			1.00
Ⅰa	67 (54.03)	169 (68.15)		15 (1.00)	30 (1.00)		67 (1.00)	15 (1.00)	
Ⅰb	29 (23.39)	35 (14.11)		0 (0.00)	0 (0.00)		0 (0.00)	0 (0.00)	
Ⅱa	8 (6.45)	13 (5.24)		0 (0.00)	0 (0.00)		0 (0.00)	0 (0.00)	
Ⅱb	9 (7.26)	11 (4.44)		0 (0.00)	0 (0.00)		0 (0.00)	0 (0.00)	
Ⅲa	11 (8.87)	18 (7.26)		0 (0.00)	0 (0.00)		0 (0.00)	0 (0.00)	
Ⅲb	0 (0.00)	2 (0.80)		0 (0.00)	0 (0.00)		0 (0.00)	0 (0.00)	

### 2.4 术中、术后观察项目

术中记录手术时间、出血量、淋巴结清扫站数及个数，比较发现肺叶切除与肺段切除的高龄患者与非高龄患者的差异不具有统计学意义(*P* > 0.05)，而对高龄患者行肺叶切除较肺段切除能清扫更多站纵隔淋巴结，清扫的个数也相对较多。术后住院期间记录各组的胸管放置时间、总引流量、术后住院时间、术后视觉模拟评分法(visual analogue scale, VAS)疼痛评分及住院总费用，均无明显的统计学差异(*P* > 0.05)。虽然总体术后并发症发生率上，高龄肺叶切除及肺段切除两组无明显统计学差异(*P* > 0.05)，但是在房颤和双下肢静脉血栓发生率上两组存在统计学差异(*P* < 0.05)。具体如表 6。

**6:** 术中、术后观察项目情况[*n*(%)] Intraperative and postoperative data [*n*(%)]

	SP VATS-L (*n*=372)		*P*	SP VATS-S (*n*=45)		*P*	SP VATS-E (*n*=139)		*P*
	E (*n*=124)	NE (*n*=248)		E (*n*=15)	NE (*n*=30)		L (*n*=67)	S (*n*=15)	
Operation time (Mean±SD, min)	191.75±5.09	181.81±2.93	0.07	211.93±17.41	202.77±8.58	0.60	194.81±8.09	211.93±17.41	0.37
Intraoperative blood loss (Mean±SD, mL)	75.60±49.43	69.42±47.15	0.24	94.67±18.62	66.83±8.59	0.13	74.55±6.19	94.67±18.62	0.20
Numbers of mediastinal nodal stations (Mean±SD)	7.72±0.15	7.52±0.10	0.28	5.60±0.35	2.37±0.33	0.66	7.61±0.21	5.60±0.35	0.00
Numbers of dissected lymph nodes	20.91±0.68	19.87±0.56	0.26	15.40±2.64	11.57±1.59	0.63	20.39±0.97	15.40±2.64	0.00
Postoperative VAS pain score	3.02±0.11	3.10±0.08	0.55	2.87±0.26	2.93±0.24	0.86	3.03±0.15	2.87±0.26	0.63
Postoperative complications	9 (7.26%)	17 (6.85%)	0.85	3 (20.00%)	3 (10.00%)	0.35	4 (5.97%)	3 (20.00%)	0.20
Lung infection	5 (4.03%)	5 (2.02%)	0.08	2 (13.33%)	1 (3.33%)	0.21	4 (5.97%)	2 (13.33%)	0.32
Postoperative atrial fibrillation	3 (2.42%)	4 (1.61%)	0.59	1 (6.67%)	0 (0.00%)	0.15	0 (0.00%)	1 (6.67%)	0.03
Vein thrombosis of lower limbs	1 (0.81%)	1 (0.40%)	0.62	1 (6.67%)	1 (3.33%)	0.61	0 (0.00%)	1 (6.67%)	0.03
Pulmonary embolism	1 (0.81%)	0 (0.00%)	0.16	0 (0.00%)	0 (0.00%)	1	0 (0.00%)	0 (0.00%)	1.00
Chylothorax	0 (0.00%)	3 (1.21%)	0.22	0 (0.00%)	0 (0.00%)	1	0 (0.00%)	0 (0.00%)	1.00
Postoperative leakage	3 (2.42%)	6 (2.42%)	1.00	0 (0.00%)	1 (3.33%)	0.48	0 (0.00%)	0 (0.00%)	1.00
Postoperative drainage duration (Mean±SD, d)	6.65±0.74	5.73±0.94	0.52	5.00±0.74	4.17±0.47	0.33	7.00±1.31	5.00±0.74	0.48
Postoperative drainage volume(Mean±SD, mL)	1, 145.60±103.13	838.02±67.02	0.06	853.53±177.04	572.57±135.40	0.23	1, 150.15±140.02	853.53±177.04	0.34
Duration of postoperative hospital stay (Mean±SD, d)	3.02±1.22	3.10±1.30	0.55	5.04±1.30	4.70±0.48	0.24	3.18±1.32	5.04±1.30	0.96
Costs (Mean±SD, thousands yuan)	61.51±4.93	54.92±0.97	0.08	7.21±5.23	5.91±1.74	0.06	70.06±5.23	61.20±5.22	0.34
VAS: visual analogue scale.									

### 2.5 术后随访

417例患者术后采用门诊随访及电话随访的方式随访94天-815天(平均403.5天)，截止末次随访时间，未发现肿瘤局部复发及肿瘤相关性死亡。

## 3 讨论

随着人口老龄化的逐步加重及肺癌患病率的逐渐提高，高龄肺癌患者的手术治疗也受到关注。目前在人类学研究上并没有明确定义“高龄”的范围，因此，“高龄”被赋予具有较多的“子项”——范围均在65岁-80岁，较为不明确。查阅国内外相关文献可发现，“高龄”主要有被定义为4个年龄节点——65岁、70岁、75岁或80岁^[15]^。通过相关数据库查询国内外研究“高龄”的文献，可发现有较多类似将“高龄”入组标准定义为“65岁及以上”患者的文献，其中不乏本学科领域的相关研究^[16-20]^。

高龄患者体质差、免疫力差，时间较长、创伤较大的手术方式无疑对其治疗效果不佳，生存率无可保障。本研究结果就发现行单孔胸腔镜肺叶切除或肺段切除的高龄患者多术前合并有高血压病或糖尿病，两者兼有的有14例，所以我科在术前针对高血压病和糖尿病患者，会予以严格的血压、血糖监测，低盐、糖尿病饮食，请相关科室协助诊疗，控制血压及血糖接近正常水平，以降低手术麻醉风险，降低术后伤口感染、不愈合发生率等。但是高龄并不是手术禁忌，若患者心肺功能可耐受全麻手术，手术疗效还是可以肯定的。行肺叶切除术的肺功能检查最低限度为一秒用力呼气容积(forced expiratory volume in one second, FEV_1_) > 1.0 L、每分钟最大通气量(maximal voluntary ventilation, MVV)% > 40%^[21]^，入组本次研究的高龄肺癌患者FEV_1_在1.27 L-4.80 L，MVV%在59.50%-98.60%，均可耐受手术，且肺叶切除高龄组与肺段切除高龄组在术前肺功能上无明显差异(*P* > 0.05)。曾有学者研究过，高龄肺癌患者行手术治疗后的2年生存率为35.6%，而不接受手术治疗的仅为6%。本研究对所有入组患者均进行了随访，截至随访时间前尚未发现行手术治疗后的高龄患者出现肿瘤局部复发及肿瘤相关性死亡。

本研究中，行单孔胸腔镜下肺叶切除或肺段切除的高龄肺癌患者与非高龄肺癌患者在手术时间、术中出血量及纵隔淋巴结清扫情况上无明显差异(*P* > 0.05)，能获得类似的手术效果。肺叶切除与肺段切除的高龄肺癌患者在手术时间及术中出血量上也无明显差异(*P* > 0.05)，且与既往国内外相关报道^[22-25]^类似。只是在手术时间的绝对值上较其长，造成这种结果的原因是高龄NSCLC患者自身体质较差，所以麻醉的创伤对于这类病人也相当可观，遂将麻醉开始作为手术时间的开端，而麻醉苏醒作为手术时间的结束。目前研究认为，纵隔淋巴结清扫范围应包括3组或3组以上的纵隔淋巴结，其中需包含隆突下淋巴结(第7组纵隔淋巴结)，清扫数目应不少于10枚^[26]^。本研究中，高龄肺癌患者行肺叶切除清扫纵隔淋巴结(7.61±0.21)组，清扫纵隔淋巴结(20.39±0.97)枚，高龄肺癌患者行肺段切除清扫纵隔淋巴结(5.60±0.35)组，清扫纵隔淋巴结(15.40±2.64)枚，两组在清扫纵隔淋巴结站数及数目上的差异有统计学意义(*P* < 0.05)，考虑因为肺段切除范围小于肺叶，使得段间及相邻段内淋巴结(第12组-14组)清扫的范围缩小，且我科行肺段切除时术中肺门或纵隔淋巴结冰冻病理阴性，常规多行纵隔淋巴结采样，若阳性则转为肺叶切除，常规行纵隔淋巴结清扫。

术后常规病理中，肺段切除高龄组与非高龄组在肿瘤最大径、病理类型和病理分期上的无统计学差异(*P* > 0.05)；肺叶切除高龄组与非高龄组在肿瘤最大径上差异无统计学意义(*P* > 0.05)，但是在病理组织类型上非高龄组较高龄组微浸润性腺癌少，浸润性腺癌和鳞癌多，但是两者差异在病理分期上并无明显统计学差异(*P* > 0.05)，考虑与高龄患者机体代谢能力下降，癌组织的活性较低，肿瘤的侵袭和转移能力较低有关。肺段切除高龄组的肿瘤最大径较肺叶切除高龄组小，这与既往研究^[27, 28]^相仿。在病理类型上，肺叶切除高龄组较肺段切除高龄组组织恶性度更高，但是术后VAS疼痛评分、胸管放置时间、总引流量和术后住院时间上无统计学差异(*P* > 0.05)，考虑因肺叶切除手术指征较肺段切除适应范围更广泛，回顾性研究肺段切除病例选择更加局限，这可能会影响最终数据，使研究结论产生偏移。因此，笔者认为肿瘤大小并不是治疗效果的决定因素，应期待更多相关的前瞻性研究加以证实。

肺叶切除高龄组术后并发症发生率为5.97%，肺段切除高龄组术后并发症发生率为20.00%，差异无统计学意义(*P* > 0.05)。但是在术后房颤和双下肢静脉血栓发生率上存在统计学差异(*P* < 0.05)，就绝对数据而言，术后发生房颤和双下肢静脉血栓者均为行肺段切除术患者，考虑是由于肺段切除的高龄患者在年龄上较肺叶切除者大，术前合并症多，以至于术后并发症的绝对值较高，且术后房颤和术后双下肢静脉血栓患者仅各为1例，存在较大的统计学偏移，笔者认为该项研究结果无特殊意义，有待进一步前瞻性或大样本研究予以证明。

曾有国内外学者^[28, 29]^研究非单孔全胸腔镜下解剖性肺段切除和肺叶切除的疗效报道：肺段切除术的手术时间更长､纵隔淋巴结清扫的站数和数目更少､术后引流量更少､术后引流天数更短､总住院费用更多。本研究中，肺叶切除高龄组与肺段切除高龄组所研究的数据虽然在绝对值上与其结果相似，但是手术时间、总引流量、胸管放置时间、总住院费用上两者并无统计学差异(*P* > 0.05)。

我国学者赵纯^[30]^对于非单孔全胸腔镜下解剖性肺段切除和肺叶切除的疗效研究结果报道：总引流量[肺段组：(654.71±302.97)mL，肺叶组：(789.09±400.26)mL]，胸管放置时间[肺段组：(3.89±1.20)d，肺叶组：(4.03±1.31)d]。本研究结果：总引流量[肺段高龄组：(853.53±177.04)mL，肺叶高龄组：(1, 150.15±140.02)mL]，胸管放置时间[肺段高龄组：(5.00±0.74)d，肺叶高龄组：(7.00±1.31)d]。考虑为我科术中留置的引流管为1根28 F闭式引流管和一根艾贝尔超细引流管，术后疼痛主要由胸痛28 F闭式引流管引起，所以较早拔除，留置艾贝尔超细引流管，管身柔软，在重力作用及肺挤压下其又位于胸膜腔最低点，实时引流更充分，且超细引流管内壁光滑，抗血液凝集能力强，不易堵管，既往有学者^[31]^做过类似比较研究。

在住院总费用因素上，高龄NSCLC患者无论行哪种手术方式差异无统计学意义，但是在绝对值上肺叶切除高龄组住院费用较肺段切除高龄组费用高，其原因主要是我科行精准肺段切除时所用腔镜直线切割缝合器钉仓数目较少，在处理段间平面的时候多使用超声刀离断，只是这对术者的技术要求更高。

综上所述，单孔胸腔镜肺叶切除术或肺段切除术应用于高龄NSCLC患者在术后近期内可获得类似非高龄NSCLC患者的疗效。结合高龄患者自身特点而言——心肺功能减弱，对手术耐受力低，术后容易发生呼吸、循环系统的并发症，因为肺段切除较肺叶切除保留更多正常肺组织，术式更加微创精准，术后有再次手术的机会，对合适的高龄病例行肺段切除可获得与肺叶切除相类似的近期疗效，具有推荐性。
